# Syringomatous dermatitis: a myth or an existing entity?

**DOI:** 10.1007/s00403-023-02537-1

**Published:** 2023-02-13

**Authors:** Hussein M. M. Hassab-El-Naby, Ahmed H. Nouh

**Affiliations:** Department of Dermatology, Venereology and Andrology, Faculty of Medicine, Al-Azhar University, 91, El Hegaz Street, Heliopolis, Cairo, 11757 Cairo Governorate Egypt

**Keywords:** Classic syringoma, Eruptive syringoma, Reactive syringomatous proliferation

## Abstract

Syringoma is rare disease that in classical variant occurs mainly on lower eyelids. In previously published researches, there is increasing evidence that eruptive syringomas must be discussed as an inflammatory dermal reaction pattern. And there was a proposal to change a name from eruptive syringoma to reactive syringomatous proliferation in appropriate cases. We conduct retrospective study on histopathological archived material to study the histopathological findings in cases of eruptive syringomas and correlate it with hypothesis that eruptive syringomas is not true adnexal neoplasms “de novo” but a hyperplastic response of the acrosyringium to an inflammatory process.

According to obtained data and literature correlation, we can conclude that there is apparent diversity in eruptive syringomas. Part of cases can be calculated as neoplastic lesions arising “de novo,” and another part as reactive syringomatous proliferation due to preceding cutaneous inflammatory process. Thus, term “eruptive syringoma” may be changed in appropriate cases to a “reactive syringomatous proliferation.”

Clinical variants of eruptive syringoma with evidence of underlying inflammatory process may be more responsive on types of treatments used for inflammatory disorders. Thus, more global clinicopathological correlative researches should be conducted and classification with terminology should be appropriately changed.

## Introduction

Syringoma has traditionally been described as a benign tumor derived from the intraepidermal part of eccrine sweat ducts [1]. A classical variant of syringoma is clinically presented with asymptomatic soft skin-covered yellowish multiple (sometimes solitary) symmetrical bilateral papules, which are situated mainly on the lower eyelids. The disease is rare, and the lesions are harmless for patients having only cosmetical drawbacks. Histopathologically, they consist of a small dermal collection of obvious sweat ducts, which are of “tadpole” shape, in a dense sclerotic background. They are usually situated in the superficial dermis, although there are some observed cases when they extend into the deep dermis. They do not infiltrate into subcutis or deeper tissues [2].

The term “eruptive syringoma” is usually used to describe successive crops of syringomas in any other location rather than facial [3]. There are several reports supporting the hypothesis that pathomechanisms and the genuine neoplastic character of eruptive syringomas are controversial [1, 4–8]. Most probably they occur as the consequence of chronic inflammatory processes of the skin involving adnexal structures. The influence of hormones on the development of syringomas is discussed due to the observation that syringomas have a higher incidence in females and frequently occur around and during puberty.

There is increasing evidence [6, 9–11] that the occurrence of eruptive syringomas or coincidental findings of these neoplasms in skin biopsies from scarring alopecia, melanocytic lesions, prurigo nodularis, and contact dermatitis must be discussed as an inflammatory dermal reaction pattern concerning the acrosyringium, the so-called reactive syringomatous proliferation due to distinct inflammatory or hormonal stimuli. So, there was a proposal to change the name eruptive syringoma to reactive syringomatous proliferation in appropriate cases.

## Aim of this work

To study the histopathological findings in cases of eruptive syringomas and correlate them with hypothesis that eruptive syringomas are not true adnexal neoplasms “de novo” but a hyperplastic response of the acrosyringium to an inflammatory process.

## Materials and methods

The research was retrospective cross-sectional single-center blinded study of possibility of histopathological differentiation between classic syringoma and eruptive syringoma (reactive syringomatous proliferation). The study was conducted on the database of the dermatopathology unit of Dermatology, Venereology and Andrology department of Al-Azhar University, Cairo. Tissue samples used in this study were punch biopsies handled as formalin-fixed paraffin-embedded hematoxylin- and eosin-stained histopathological microscopic sections obtained for diagnostic purposes.

Studied cases include all available samples in department collection from 2012 to 2020 with a total number of 23 samples.

Inclusion criteria:

The cases of classic and eruptive syringomas in patients of any age and sex.

Exclusion criteria:

1. The lack of clinical data
2. A bad condition of the microscopic section with inability to get a new section from paraffin blocks.

The cases of eruptive and classic syringomas were revealed from histopathological collection and revised blindly (unaware of the clinical parameter of interest) by dermatopathologist to distinguish between the cases with evidence of presence/absence of inflammatory signs.

The clinical data, archived photograph and case histories, of each case were revised blindly (unaware of the histopathological findings) by dermatologist.

The statistical analyses were performed using SPSS IBM Software version 25.0 for Windows. The clinical data of each case were correlated with their histopathological changes.

## Results

**Fig. 1:**
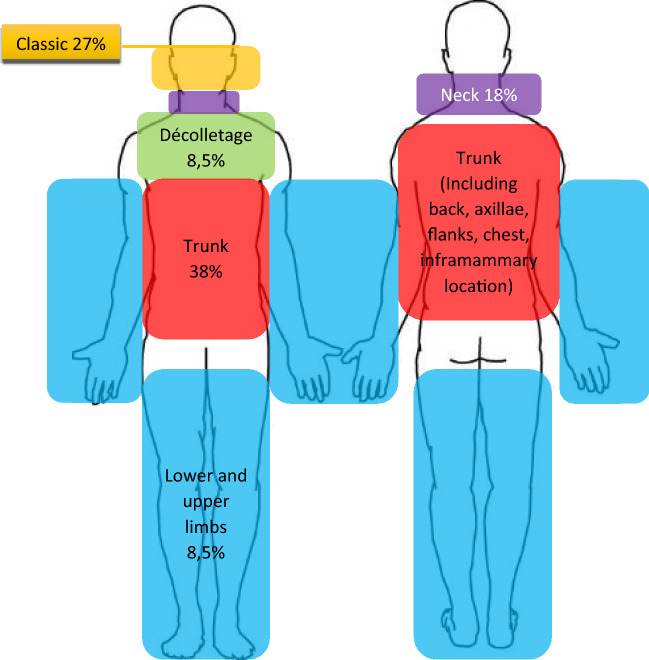
Distribution of lesions between body sites

After applying exclusion criteria, total number of complete cases was 20 (see Table 1). Six cases were males; 14 cases were females with age ranged from 10 to 53 (mean 25.3) years old. The distribution of the lesions by body sites is presented in Fig. 1. The duration of disease is ranged from 6 month to 20 years (mean 5.6 years). The distribution of the period of onset showed 2 patients in the first decade (10%), 12 patients in the second decade (60%), 4 patients in the third decade (20%), and 1 patient in fourth and fifth decades (5% each).

**Table 1:** Pivot table of clinical and histopathological data

N	Age	Gender	Clinical appearance of lesion	Pruritus	Inflammatory signs	Density of infiltrate	Milium
1	45	Male	Erythematous	Present	Positive	Dense	Absent
2	42	Female	Erythematous	Present	Positive	Dense	Absent
3	31	Female	Erythematous	Absent	Negative	Absent	Present
4	17	Female	Not erythematous	Absent	Negative	Absent	Absent
5	14	Male	Erythematous	Present	Positive	Dense	Present
6	24	Female	Not erythematous	Absent	Negative	Absent	Absent
7	24	Female	Erythematous	Present	Positive	Dense	Absent
8	22	Female	Not erythematous	Present	Negative	Absent	Absent
9	23	Male	Not erythematous	Absent	Negative	Absent	Absent
10	18	Female	Erythematous	Present	Positive	Moderate	Absent
11	21	Female	Not erythematous	Present	Negative	Absent	Absent
12	53	Female	Not erythematous	Absent	Negative	Absent	Absent
13	19	Female	Not erythematous	Present	Negative	Absent	Absent
14	19	Female	Not erythematous	Absent	Negative	Absent	Absent
15	27	Female	Not erythematous	Absent	Negative, CCS!	Absent	Absent
16	12	Male	Erythematous	Absent	Positive	Low density	Absent
17	10	Male	Erythematous	Absent	Positive	Moderate	Present
18	32	Female	Erythematous	Present	Positive	Moderate	Present
19	26	Female	Not erythematous	Present	Negative	Absent	Absent
20	27	Male	Not erythematous	Absent	Negative	Absent	Absent

*CCS* clear cell syringoma

Clinically erythematous lesions make 45% (9 cases) from total amount of the observed cases. One of which (5% from total subjects) was histopathologically negative, while others revealed inflammatory changes upon histopathological examination. From 8 positive cases (40%) with evidence of inflammatory changes, 4 were male patients and 4 were females. All these cases clinically show the erythematous color of papules. One case has lesions in décolletage, all the rest have them in trunk (including chest, abdomen, flanks, axillae, back), beside this, some cases (and all of them are females) in addition have classic syringoma around eyes. According to the patients’ case histories, pruritus was noticed in 10 cases (50%). Between erythematous lesions incidence of pruritus was twice more prevalent than in non-pruritic lesions, 6 cases (67%) versus 4 cases (33%), respectively.

Presence of lymphocytic inflammatory infiltrate was shown in all our cases with histopathological evidence of inflammatory signs 100% (*P* < 0.05 at the Fisher’s exact test).

In 4 (20% of all subjects) cases, milium was present as histopathological findings (3 cases with erythematous lesions and histopathological evidence of inflammation, and one without evidence of inflammation).

Twelve negative cases (without histopathological evidence of underlying inflammation) showed almost skin-colored lesions (9) and dusky erythematous lesions (3)—75% and 25%, respectively.

Case histories of all subjects were negative regarding using waxing/shaving, drugs or skin diseases that could explain the inflammatory origin of the lesions.

**Fig. 2:**
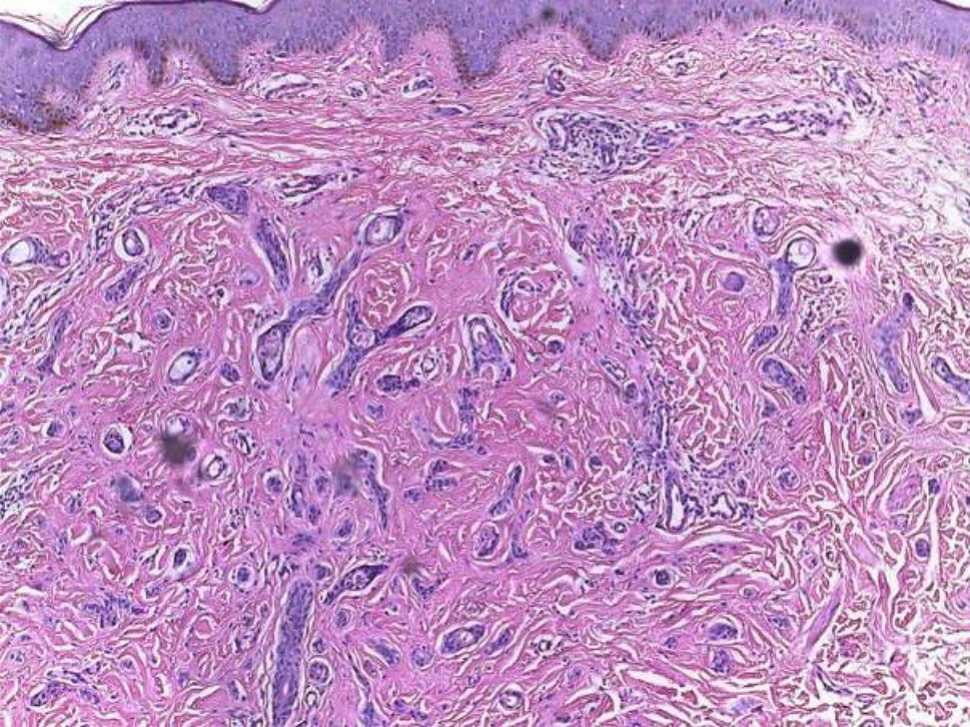
Classic changes of syringoma—comma-shaped ducts and basaloid proliferation. H&E X100

**Fig. 3:**
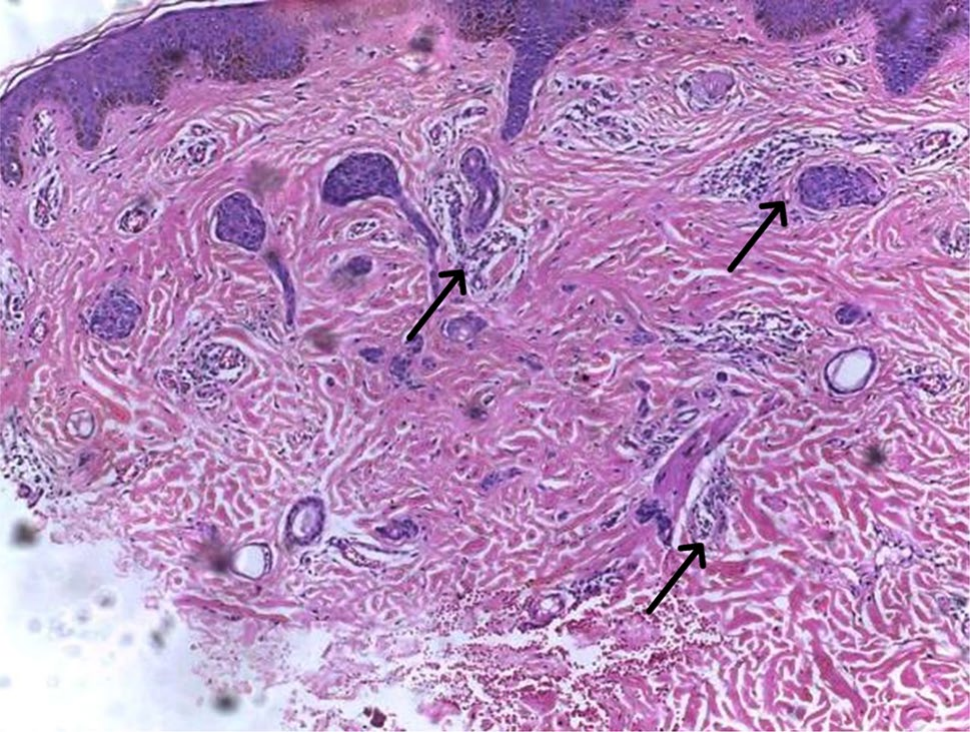
Syringoma, notice the moderately dense periductal infiltrate of lymphocytes (black arrows). H&E X100

Histopathological characteristics of lesions include acanthosis, basal hyperpigmentation, proliferation of fibrous stroma, vacuolization of inner layer of ductal cells, keratin-filled cysts (milia), tadpole appearance of ducts (coma like tails) and inflammatory infiltrate formed mainly of lymphocytes. Typical pathology is presented in Figs. 2  and 3.

**Fig. 4:**
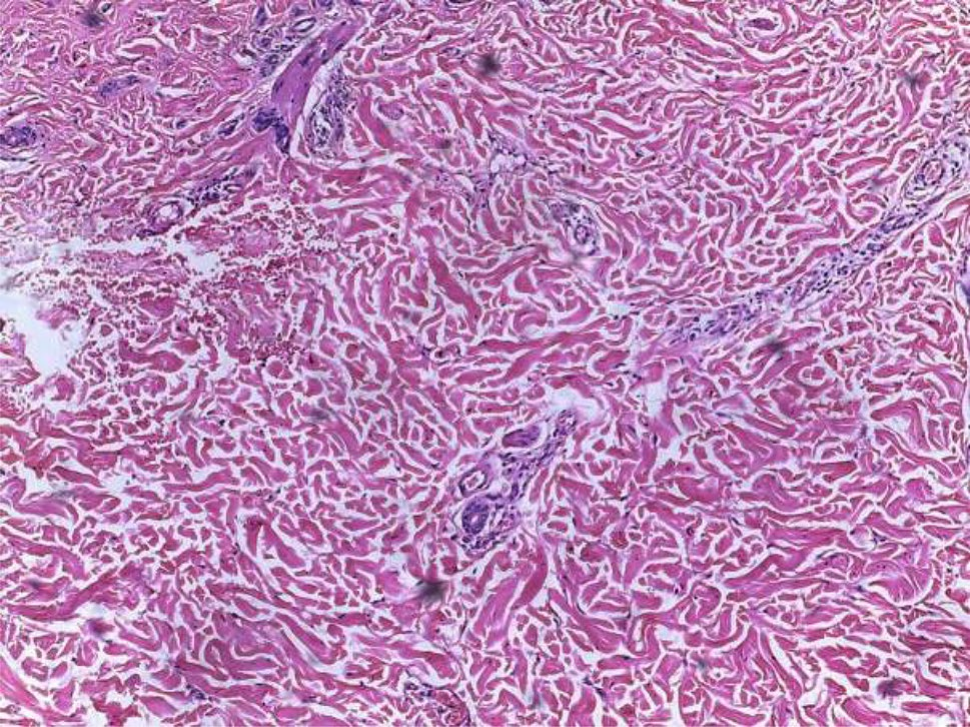
Notice the extension of lymphocytic infiltrate around deep dermal eccrine ducts. H&E X100

Statistical comparative analysis was performed between all findings. The only statistically significant correlation was found between erythematous lesions and histopathologically inflammatory signs (100%), and the occurrence of such lesions is mainly in second decade of the patient’s life (90%) [Chi-square tests, *p* = 0.033] (see Fig. 4).

## Discussion

Primary syringoma was described simultaneously by Kaposi [12] and Biesiadecki in 1872 under different name, and, later, the term “syringoma” was used by Unna in 1894. The “eruptive” variant was first described by Jaquet and Darier in 1887 [13].

The incidence of classic syringoma is around 1% in world population and appears to be higher in Asians and Africans [10, 14]. The eruptive variant is even more rare [9, 15].

The literature search by relevant keywords reveals 64 cases of eruptive syringomas reported before 2001 [10] and 53 more cases in a period of 2001–2020.

The currently used clinical classification proposed in 1987 by Friedman and Butler's classifies syringomas into four subtypes: a localized form, a familial form, a form associated with Down's syndrome and a generalized form that encompasses multiple and eruptive syringomas [16].

The eruptive form of syringoma usually manifests itself with multiple 2-to-5 mm monomorphic papules that are shiny, firm, flat-topped and range in color between skin-colored, slightly yellowish or hyperpigmented [1, 3, 7, 10, 19, 20]. The clinical presentation of our cases of eruptive syringomas is obviously divided into two types, first skin-colored to yellowish and second–discolored (erythematous, hyperpigmented). Exactly this clinical presentation (erythematous discoloration of lesions) has led us to the idea to conduct this research to correlate it with histopathological evidence of inflammatory process.

Most authors who support the theory of not a “neoplastic de novo” origin of eruptive syringoma noticed that in many cases identification of preceding inflammatory process does not seem possible [1, 9, 17, 18]. We also do not found any significant evidence of inflammatory disturbances in history of subjects, and even pruritus (as sign of inflammation) was not present in all cases.

For the definitive diagnosis of syringoma, histopathological examination is mandatory. The histopathologic features of syringomas are distinct with some intriguing variations, e.g., clear-cell or milium-like variants [9]. The hematoxylin–eosin stain shows mainly normal epidermis and presence of multiple small ducts and epithelial cords within the dermis. The ducts are lined by one or two rows of flattened polygonal epithelial cells, the outer layer bulging outward to create a comma-like tail. Different severity of fibroplasia of surrounding stroma frequently observed [9, 21]. Furthermore, dilated ducts with eosinophilic secretion may be seen [10, 14, 18]. In cases with clinically seen erythematous lesions, we can see marked lymphocytic infiltrate involving the intraepidermal and upper dermal portions of the eccrine gland [1]. Presence of such infiltrate was shown in all our cases with histopathological evidence of inflammatory signs. Milia can be present in samples [16, 22] and may point to inflammatory status. In our study were 4 such cases, 3 of which were histopathologically positive of inflammatory signs.

The pathogenesis of eruptive syringomas still remains unclear. Few publications outlined the association of eruptive syringomas and neoplasms or diabetes mellitus without pathogenic relationship [10]. Some authors still assume that syringoma is a tumor of sporadic origin or hereditary etiology [23]. Indeed, familial syringoma (autosomal dominant inheritance) has been described and may be underestimated in prevalence [24–27].

Syringomatous proliferation and syringoma-like changes have been reported in association with diffuse alopecia, alopecia areata, melanocytic nevi, prurigo nodularis, milia^1^, which are suggestive of reactive syringomatous proliferation as an answer to the inflammatory cutaneous process. Some case reports noted the drug-induced syringomatous proliferation. Such drug includes oral contraceptives [6, 7, 9, 11], antiepileptics [28], etc. Eruptive syringomas are only associated with one genodermatosis—the Nicolau–Balus syndrome, which include syringomas, milia and atrophoderma vermiculata [29].

The disease is not self-limiting [10] and requires treatment, which is often frustrating, due to the slow growth and multicentric occurrence and the risk of scarring due to therapeutic interventions [9]. Most helpful and popular treatment modalities include topical or systemic retinoids, laser ablation, cryosurgery, dermabrasion, and chemical peelings. There are certain reports of successful treatments of eruptive syringoma with 1% topical atropine [30–33]. However, all therapeutic options bear the risk of recurrence, and general aesthetic effect is usually not satisfactory enough.

## Conclusion

According to the available data in the literature and our clinical/histopathological experience there is apparent diversity in eruptive syringomas. Some cases can be considered as neoplastic lesions arising “de novo”, and others as reactive syringomatous proliferation due to the preceding cutaneous inflammatory process.

Thus, the term “eruptive syringoma” may be added to classification and changed in appropriate cases to a “reactive syringomatous proliferation” proposed by Cornelia and co-authors in 2009 [13].

The clinical variants of eruptive syringoma with evidence of the underlying inflammatory process may be more responsive to the types of treatments used for inflammatory disorders. Thus, more global clinicopathological correlative researches should be conducted and classification with terminology should be changed appropriately.

## Data Availability

The data that support the findings of this study are available on request from the corresponding author.
